# Mechanism of miR-132-3p Promoting Neuroinflammation and Dopaminergic Neurodegeneration in Parkinson’s Disease

**DOI:** 10.1523/ENEURO.0393-21.2021

**Published:** 2022-01-25

**Authors:** Xin Gong, Mengyi Huang, Lei Chen

**Affiliations:** Department of Neurosurgery, Hunan Provincial People’s Hospital, The First Affiliated Hospital of Hunan Normal University, Changsha, Hunan 410005, People’s Republic of China

**Keywords:** Parkinson’s disease, MiR-132-3p, neuroinflammation, dopaminergic neuron, GLRX, MPTP

## Abstract

The major pathology in Parkinson’s disease (PD) is neuron injury induced by degeneration of dopaminergic neurons and the activation of microglial cells. The objective of this study is to determine the effect and mechanism of miR-132-3p in regulating neuroinflammation and the degeneration of dopaminergic neuron in PD. The expressions of miR-132-3p in brain tissues of PD patients, lipopolysaccharide (LPS)-induced BV-2 cells and 1-methyl-4-phenyl-1,2,3,6-tetrahydropyridine (MPTP)-induced PD mouse models were detected. The effect of miR-132-3p and GLRX in cell viability, apoptosis and inflammation was verified in BV-2 cells. The activation of Iba1 in substantia nigra pars compacta (SNc) and the loss of tyrosine hydroxylase were detected in PD mouse models and the mobility of mouse models was assessed as well. The targeting relationship between miR-132-3p and GLRX was confirmed by RNA immunoprecipitation (RIP) and dual luciferase reporter gene assay. Elevated expression of miR-132-3p and decreased expression of GLRX were found in PD patients and cells models. Overexpression of miR-132-3p can induce activation of microglial cells, which can be reversed by GLRX overexpression. Collected evidence in both cell model and mouse models showed the effect of miR-132-3p in enhancing the activation of microglial cells and the loss of microglia cells, which was achieved by mediating GLRX.

## Visual Abstract

**Figure:**
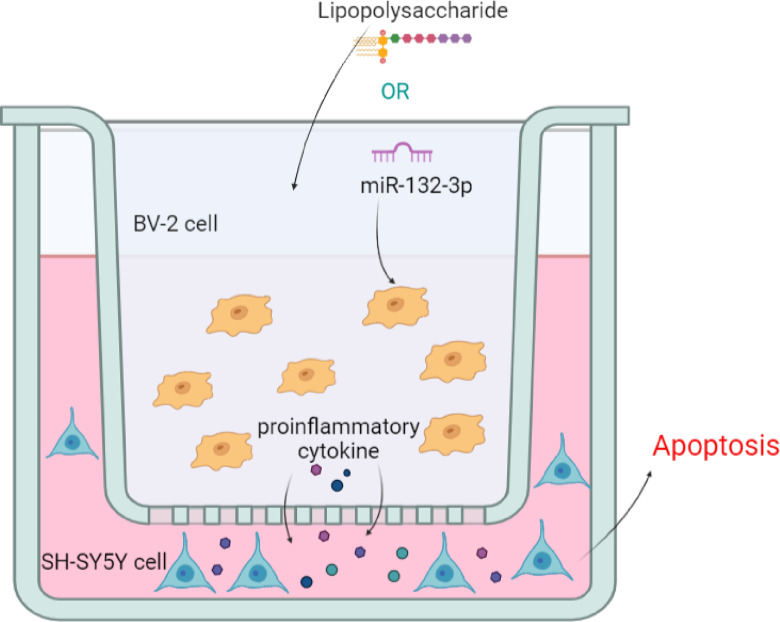

## Significance Statement

The purpose of this study is to explore the possible effect and mechanism of miR-132-3p/GLRX on neuroinflammation and the degeneration of dopaminergic neuron in Parkinson’s disease (PD). This is important as no effective treatment is available to cure PD and better understanding on how neuroinflammation and degeneration of dopaminergic neurons was regulated in PD will facilitate the proposal of therapeutic strategy. Future study should further validate the role and mechanism of miR-132-3p in regulating PD through GLRX before miR-132-3p can be proposed as a novel therapeutic target.

## Introduction

Parkinson’s disease (PD) is a neurodegenerative disease in elderly caused by degeneration of dopaminergic neurons mainly in the substantia nigra pars compacta (SNc), a region of the midbrain (Sveinbjornsdottir, 2016; Alizadeh et al., 2019). Severe locomotor deficit, such as freezing of gait, is a typical phenomenon of PD, which refers to intermittent walking disturbance during walk initiation and turning (Strubberg and Madison, 2017). The etiology of PD is probably multifactorial and currently there is no available treatment that can attenuate the neurodegenerative process of the disease. Therefore, a clearer understanding of mechanizing driving PD progression would be beneficial for the proposal of therapeutic approach. A variety of molecular mechanisms that likely contribute to neuronal cell death have been described previously, including α-synuclein aggregation, mitochondrial dysfunction and noxious oxidant stress (Milanese et al., 2021). In addition, neuroinflammation is one of the hallmarks of PD and may induce the degeneration of midbrain dopamine neurons (Krashia et al., 2019). Therefore, therapeutic intervention would be a potential strategy to alleviate the progression of PD by interfering with neuroinflammation and degeneration of dopaminergic neurons.

MicroRNA (miRNA) is an endogenic RNA comprised of 21–24 nt, which controls gene transcription in combination with the 3′-untranslated region (UTR) of multiple targeted mRNAs (Li et al., 2017; Angelopoulou et al., 2019). About 1900 miRNAs that can be encoded by human genome (Strubberg and Madison, 2017). Among these miRNAs, *miR-132* has been frequently mentioned in many researches for its increased expression in neurons and for its implication in various neurodegenerative disorders. For example, the enhanced expression of *miR-132-3p* is related to chronic neuropathic pain (Leinders et al., 2016). *MiR-132*/Nurr1 axis was reported to have certain relationship with PD progression (Yang et al., 2019). Moreover, neuronal inflammation-induced epilepsy may be attenuated by *miR-132* by targeting TRAF6, along with inactivation of NF-κB and MEK/ERK pathways (Ji et al., 2018). *MiR-132* is positively associated with dopaminergic neuronal death (Qazi et al., 2021). Evidence in previous study pointed out that microglial cells medicated neuroinflammation triggered the cascade of inflammatory events leading to neuronal degeneration (Hirsch and Hunot, 2009; Bassani et al., 2015; Ye et al., 2018). However, it remains unclear how *miR-132-3p* is involved in neuroinflammation and dopaminergic neurodegeneration in PD.

GLRX is a small protein that catalyzes the glutathione-dependent disulfide oxidoreduction reactions in a coupled system (Wang et al., 2019). In models of PD, deficiency of GLRX aggravates neurodegeneration (Johnson et al., 2015). Meanwhile, previous study addressed that suppression of GLRX contributes to PD-relevant motor deficits and dopaminergic degeneration in mice (Verma et al., 2020). Therefore, we speculated GLRX also has certain role to play in neuroinflammation and dopaminergic neurodegeneration in PD. Online software predicted that *miR-132-3p* was identified as an upstream regulatory factor of GLRX. In this regard, this study aims to investigate the mechanism by which *miR-132-3p* regulates neuroinflammation and dopaminergic neuron degeneration in PD. Hence, exploring the interactions between *miR-132-3p*/GLRX, dopaminergic neurodegeneration and neuroinflammation may be of great importance for the proposal of a latent therapeutic alternative for PD.

The main aims of the present study were to determine (1) whether *miR-132-3p* expression level is significantly altered in patients with PD as compared with healthy controls; (2) whether *miR-132-3p* is responsible for microglial activation and neuronal injury; (3) whether *miR-132-3p* affects the dopaminergic neuron degeneration and neuroinflammation in PD mouse models; and (4) whether *miR-132-3p* intensifies PD by inhibiting GLRX.

## Materials and Methods

### Collection of clinical brain tissues

The study was conducted according to the Declaration of Helsinki. The study protocol concerning human was approved by the Ethics Committee of Hunan Provincial People’s Hospital (No. 202004), and written informed consent from family members of included subjects was obtained. This study was not preregistered and no sample calculation was performed. After death, the midbrain tissues were obtained from five patients with PD (three males and two females, 55.8 ± 7.09 years old) and five healthy controls (three males and two females, 59.8 ± 8.07 years old) matched for age. The brain tissues were collected and stored in liquid nitrogen, in which the total RNA and total protein were stored at −80°C refrigerator. The diagnosis of PD was performed by at least two or more experienced neurologists based on the clinical diagnostic criteria proposed by International Parkinson and Movement Disorder Society (Postuma et al., 2018). The included PD patients were excluded from secondary PD, tumor, or metabolic disturbance. The healthy controls were excluded from disorders related to nervous system.

### Cell culture

The BV-2 microglial cells, human neuroblastoma cell line SH-SY5Y and human embryonic kidney (HEK)293T cells were supplied by American type Culture Collection (ATCC). The BV-2 and HEK293T cells were soaked in DMEM (Invitrogen), and SH-SY5Y cells were immersed in DMEM/Nutrient Mixture F-12 (DMEM/F12, Invitrogen). The culture medium contained 10% FBS, 100 U/ml penicillin, and 100 mg/ml streptomycin. Cell culture was kept at 37°C in a humidified atmosphere containing 5% CO_2_. The inflammation of BV-2 cells was induced by 0.1 μg/ml lipopolysaccharide (LPS) for 24 h. The cell passage of cell lines shall not exceed 10 times.

### Cell transfection

The *miR-132-3p* mimic (miRNA mimic refers to a sequence that can simulate specific endogenous miRNA), mimic negative control (NC), *miR-132-3p* inhibitor (miRNA inhibitor refers to a sequence that can interfere with miRNA), inhibitor NC, overexpressing GLRX (GLRX) and pcDNA3.1 were synthesized and purchased from GenePharma. Cell transfection was conducted by using the Lipofectamine 3000 reagent (Invitrogen). The transfection dose of overexpression plasmids was 2 μg, and the dose of mimic and inhibitor was 50 nm. Cells were treated by LPS for 24 h before following experiments were conducted.

### qRT-PCR

TRIzol (Invitrogen) was employed to extract the total RNA of tissues or cells, and the reverse transcription was conducted by using the reverse transcription kit (TaKaRa). The expression of gene was quantitated by LightCycler 480 fluorescence quantitative PCR instrument (Roche), and reaction condition was instructed by the fluorescence quantitative PCR kit (SYBR Green Mix, Roche Diagnostics). The thermal cycle parameters were 95°C for 10 s, followed by 45 cycles of 95°C for 5 s, 60°C for 10 s, and 72°C for 10 s. A final extension was conducted at 72°C for 5 min. The quantification of mRNA was normalized to β-actin and miRNA to U6. The fold changes were calculated by the 2^-ΔΔCt^ method. The formula is as follows: ΔΔCt = [Ct_(target gene)_ – Ct_(reference gene)_]_experimental group_ – [Ct_(target gene)_ – Ct_(reference gene)_] _control group_. All primers are shown in Table 1.

**Table 1:** Primer sequence information

Name of primer	Sequences
U6-F	CTCGCTTCGGCAGCACA
U6-R	AACGCTTCACGAATTTGCGT
miR-132-3p -F	GCAACGTAACAGTCTACAGCC
miR-132-3p -R	CCAGTGCAGGGTCCGAGGTA
β-Actin-F	TGTACCCAGGCATTGCTGAC
β-Actin-R	AACGCAGCTCAGTAACAGTCC
GLRX-F	AGTTATAAAAGGGGTGGCAGAGT
GLRX-R	CCCCATGGTTAGGGGCAAAT
TNF-α-F	AGGCACTCCCCCAAAAGATG
TNF-α-R	CCACTTGGTGGTTTGTGAGTG
IL-1β-F	TGCCACCTTTTGACAGTGATG
IL-1β-R	AAGGTCCACGGGAAAGACAC
IL-6-F	CAACGATGATGCACTTGCAGA
IL-6-R	TGTGACTCCAGCTTATCTCTTGG

F: forward primer; R: reverse primer.

**Table 2:** Abbreviation list

Abbreviations	Full names
PD	Parkinson’s disease
GLRX	Glutaredoxin
LPS	Lipopolysaccharide
qRT-RCR	Quantitative real-time polymerase chain reaction
ELISA	Enzyme-linked immunosorbent assay
RIP	RNA immunoprecipitation
MPTP	1-Methyl-4-phenyl-1, 2, 3, 6-tetrahydropyridine
SNc	Substantia nigra compacta
TH	Tyrosine hydroxylase
DMEM	Dulbecco’s modified eagle medium
PBS	Pphosphate buffer
Ago2	Argonaute 2
FBS	Fetal bovine serum
FITC	Fluorescein isothiocyanate
PI	Propidium iodide
FISH	Fluorescence *in situ* hybridization
RRID	Research Resource Identifier
NC	negative control

**Table 3:** Reagents and materials

Names	RRIDs or catalog number
BV-2 cell	YB-ATCC-4255, ATCC
SH-SY5Y cell	RRID:CVCL_0019
HEK293T cell	RRID:CVCL_0063
DMEM	11054001, Gibco
DMEM/F12	11330107, Gibco
FBS	16140, Invitrogen
Penicillin/streptomycin	15140148, Invitrogen
LPS	L-4391, Sigma-Aldrich
Lipofectamine 3000	L3000150, Invitrogen
TRIzol	15596026, Invitrogen
Reverse transcription kit	6210A, TaKaRa
SYBR Green Mix	2015099, Roche
RIPA lysis buffer	P0013C, Beyotime
BCA kit	P0012, Beyotime
Protein loading buffer	P0015A, Beyotime
β-Actin antibody	RRID:AB_306371
GLRX antibody	RRID:AB_880242
Film development kit	P0019, Beyotime
TNF-α	DY410, R&D
IL-1β	SMLB00C, R&D
IL-6	SM6000B, R&D
CCK-8 kit	CK04, Dojindo
Annexin V-FITC cell apoptosis kit	C1062L, Beyotime
PBS	C0221A, Beyotime
A + G beads	P2108, Beyotime
Ago2 antibody	RRID:AB_867543
IgG antibody	RRID:AB_2687931
pGL3-Promoter	E1761, Promega
pRL-TK	E2241, Promega
Dual-Luciferase Reporter Assay System	E1910, Promega
MPTP	M0896, Sigma-Aldrich
4% paraformaldehyde	P0099, Beyotime
Proteinase K	ST535, Beyotime
Tyrosine hydroxylase antibody	RRID:AB_2801410
Iba1 antibody	RRID:AB_2636859
DAPI	C1002, Beyotime
MiRNA mimic	B02003, GenePharma
MiRNA inhibitor	B03001, GenePharma
Gene overexpression plasmid	C05001, GenePharma
MiRNA antagomir	B05001, GenePharma

**Table 4:** Statistical summary and analysis methods

Figure reported	*N*	Normal distribution	Statistic	Statistic value (df)	*p* value	Variance source	*Post hoc* test	*Post hoc p*
1*A*, PD vs control	5	Yes	Unpaired *t* test	*t*_(8)_ = 2.644	0.0295	Difference		
1*B*, PD vs control	5	Yes	Unpaired *t* test	*t*_(8)_ = 3.418	0.0091	Difference		
1*C*, PD vs control	5	Yes	Unpaired *t* test	*t*_(8)_ = 2.449	0.0400	Difference		
2*A*	3	Yes	One-way ANOVA	*F*_(3,8)_ = 41.01	<0.0001	Treatment		
2*A*, PBS vs LPS	3	Yes					Tukey	0.0025
2*A*, LPS+inhibitor NC vs LPS+ miR-132-3p inhibitor	3	Yes					Tukey	<0.0001
2*B*, TNF-α	3	Yes	One-way ANOVA	*F*_(3,8)_ = 62.48	<0.0001	Treatment		
2*B*, TNF-α, PBS vs LPS	3	Yes					Tukey	<0.0001
2*B*, TNF-α, LPS+ inhibitor NC vs LPS +miR-132-3p inhibitor	3	Yes					Tukey	0.001
2*B*, IL-1β	3	Yes	One-way ANOVA	*F*_(3,8)_ = 68.03	<0.0001	Treatment		
2*B*, IL-1β, PBS vs LPS	3	Yes					Tukey	<0.0001
2*B*, IL-1β, LPS+ inhibitor NC vs LPS +miR-132-3p inhibitor	3	Yes					Tukey	0.001
2*B*, IL-6	3	Yes	One-way ANOVA	*F*_(3,8)_ = 55.19	<0.0001	Treatment		
2*B*, IL-6, PBS vs LPS	3	Yes					Tukey	<0.0001
2*B*, IL-6, LPS+ inhibitor NC vs LPS +miR-132-3p inhibitor	3	Yes					Tukey	0.005
2*C*, TNF-α	3	Yes	One-way ANOVA	*F*_(3,8)_ = 38.98	<0.0001	Treatment		
2*C*, TNF-α, PBS vs LPS	3	Yes					Tukey	<0.0001
2*C*, TNF-α, LPS+ inhibitor NC vs LPS +miR-132-3p inhibitor	3	Yes					Tukey	0.0059
2*C*, IL-1β	3	Yes	One-way ANOVA	*F*_(3,8)_ = 70.75	<0.0001	Treatment		
2*C*, IL-1β, PBS vs LPS	3	Yes					Tukey	<0.0001
2*C*, IL-1β, LPS+ inhibitor NC vs LPS +miR-132-3p inhibitor	3	Yes					Tukey	0.001
2*C*, IL-6	3	Yes	One-way ANOVA	*F*_(3,8)_ = 86.8	<0.0001	Treatment		
2*C*, IL-6, PBS vs LPS	3	Yes					Tukey	<0.0001
2*C*, IL-6, LPS+ inhibitor NC vs LPS +miR-132-3p inhibitor	3	Yes					Tukey	0.001
3*A*	3	Yes	One-way ANOVA	*F*_(2,6)_ = 343.2	<0.0001	Treatment		
3*A*, mimic NC vs miR-132-3p mimic	3	Yes					Tukey	<0.0001
3*B*, TNF-α	3	Yes	One-way ANOVA	*F*_(2,6)_ = 47.96	0.0002	Treatment		
3*B*, TNF-α, mimic NC vs miR-132-3p mimic	3	Yes					Tukey	0.0003
3*B*, -IL-1β	3	Yes	One-way ANOVA	*F*_(2,6)_ = 51.95	0.0002	Treatment		
3*B*-IL-1β, mimic NC vs miR-132-3p mimic	3	Yes					Tukey	0.0003
3*B*, IL-6	3	Yes	One-way ANOVA	*F*_(2,6)_ = 35	0.0005	Treatment		
3*B*, IL-6, mimic NC vs miR-132-3p mimic	3	Yes					Tukey	0.0016
3*C*, TNF-α	3	Yes	One-way ANOVA	*F*_(2,6)_ = 69.99	<0.0001	Treatment		
3*C*, TNF-α, mimic NC vs miR-132-3p mimic	3	Yes					Tukey	0.0002
3*C*, IL-1β	3	Yes	One-way ANOVA	*F*_(2,6)_ = 188.5	<0.0001	Treatment		
3*C*, IL-1β, mimic NC vs miR-132-3p mimic	3	Yes					Tukey	<0.0001
3*C*, IL-6	3	Yes	One-way ANOVA	*F*_(2,6)_ = 152.1	<0.0001	Treatment		
3*C*, IL-6, mimic NC vs miR-132-3p mimic	3	Yes					Tukey	<0.0001
4*A*	3	Yes	One-way ANOVA	*F*_(3,8)_ = 68.62	<0.0001	Treatment		
4*A*, PBS vs LPS	3	Yes					Tukey	<0.0001
4*A*, LPS+inhibitor NC vs LPS+ miR-132-3p inhibitor	3	Yes					Tukey	0.0050
4*B*	3	Yes	One-way ANOVA	*F*_(3,8)_ = 114.1	<0.0001	Treatment		
4*B*, PBS vs LPS	3	Yes					Tukey	<0.0001
4*B*, LPS+inhibitor NC vs LPS+ miR-132-3p inhibitor	3	Yes					Tukey	<0.0001
4*C*	3	Yes	One-way ANOVA	*F*_(2,6)_ = 9.25	0.0147	Treatment		
4*C*, mimic NC vs miR-132-3p mimic	3	Yes					Tukey	0.0461
4*D*	3	Yes	One-way ANOVA	*F*_(2,6)_ = 17.59	0.0031	Treatment		
4*D*, mimic NC vs miR-132-3p mimic	3	Yes					Tukey	0.0040
5*A*	3	Yes	One-way ANOVA	*F*_(3,8)_ = 35.92	<0.0001	Treatment		
5*A*, inhibitor NC vs miR-132-3p inhibitor	3	Yes					Tukey	0.006
5*A*, mimic NC vs miR-132-3p mimic	3	Yes					Tukey	0.0089
5*B*	3	Yes	One-way ANOVA	*F*_(3,8)_ = 13.63	0.0005	Treatment		
5*B*, inhibitor NC vs miR-132-3p inhibitor	3	Yes					Tukey	0.01
5*B*, mimic NC vs miR-132-3p mimic	3	Yes					Tukey	0.0382
5*C*, miR-132-3p	3	Yes	One-way ANOVA	*F*_(3,8)_ = 115.1	<0.0001	Treatment		
5*C*, miR-132-3p, IgG vs Ago2	3	Yes					Tukey	<0.0001
5*C*, GLRX	3	Yes	One-way ANOVA	*F*_(3,8)_ = 126.1	<0.0001	Treatment		
5*C*, GLRX, IgG vs Ago2	3	Yes					Tukey	<0.0001
5*D*, PBS vs LPS	3	Yes	Two-way ANOVA	*F*_(2,12)_ = 12.1	0.0013	Interaction		
5*D*, PBS vs LPS	3	Yes	Two-way ANOVA	*F*_(2,12)_ = 168.8	<0.0001	Main effect		
5*F*	3	Yes	One-way ANOVA	*F*_(3,8)_ = 9.623	0.0050	Treatment		
5*F*, wt-GLRX+ mimic NC vs wt-GLRX+miR-132-3p mimic	3	Yes					Tukey	0.0044
6*A*	3	Yes	One-way ANOVA	*F*_(2,6)_ = 75.16	<0.0001	Treatment		
6*A*, mimic NC vs miR-132-3p mimic	3	Yes					Tukey	0.002
6*A*, miR-132-3p mimic vs miR-132-3p mimic+GLRX	3	Yes					Tukey	<0.0001
6*B*	3	Yes	One-way ANOVA	*F*_(2,6)_ = 36.68	0.0004	Treatment		
6*B*, mimic NC vs miR-132-3p mimic	3	Yes					Tukey	0.0165
6*B*, miR-132-3p mimic vs miR-132-3p mimic+GLRX	3	Yes					Tukey	0.004
6*C*, TNF-α	3	Yes	One-way ANOVA	*F*_(2,6)_ = 43.7	0.0003	Treatment		
6*C*, TNF-α, mimic NC vs miR-132-3p mimic	3	Yes					Tukey	0.0003
6*C*, TNF-α, miR-132-3p mimic vs miR-132-3p mimic+GLRX	3	Yes					Tukey	0.001
6*C*, IL-1β	3	Yes	One-way ANOVA	*F*_(2,6)_ = 38.76	0.0004	Treatment		
6*C*, IL-1β, mimic NC vs miR-132-3p mimic	3	Yes					Tukey	0.0004
6*C*, IL-1β, miR-132-3p mimic vs miR-132-3p mimic+GLRX	3	Yes					Tukey	0.002
6*C*, IL-6	3	Yes	One-way ANOVA	*F*_(2,6)_ = 36.31	0.0004	Treatment		
6*C*, IL-6, mimic NC vs miR-132-3p mimic	3	Yes					Tukey	0.0013
6*C*, IL-6, miR-132-3p mimic vs miR-132-3p mimic+GLRX	3	Yes					Tukey	0.0005
6*D*, TNF-α	3	Yes	One-way ANOVA	*F*_(2,6)_ = 49.81	0.0002	Treatment		
6*D*, TNF-α, mimic NC vs miR-132-3p mimic	3	Yes					Tukey	0.0002
6*D*, TNF-α, miR-132-3p mimic vs miR-132-3p mimic+GLRX	3	Yes					Tukey	0.0011
6*D*, IL-1β	3	Yes	One-way ANOVA	*F*_(2,6)_ = 131.6	<0.0001	Treatment		
6*D*, IL-1β, mimic NC vs miR-132-3p mimic	3	Yes					Tukey	<0.0001
6*D*, IL-1β, miR-132-3p mimic vs miR-132-3p mimic+GLRX	3	Yes					Tukey	<0.0001
6*D*, IL-6	3	Yes	One-way ANOVA	*F*_(2,6)_ = 65.52	<0.0001	Treatment		
6*D*, IL-6, mimic NC vs miR-132-3p mimic	3	Yes					Tukey	0.0001
6*D*, IL-6, miR-132-3p mimic vs miR-132-3p mimic+GLRX	3	Yes					Tukey	0.003
6*E*	3	Yes	One-way ANOVA	*F*_(2,6)_ = 8.975	0.0157	Treatment		
6*E*, mimic NC vs miR-132-3p mimic	3	Yes					Tukey	0.0199
6*E*, miR-132-3p mimic vs miR-132-3p mimic+GLRX	3	Yes					Tukey	0.0312
6*F*	3	Yes	One-way ANOVA	*F*_(2,6)_ = 15.91	0.004	Treatment		
6*F*, mimic NC vs miR-132-3p mimic	3	Yes					Tukey	0.0047
6*F*, miR-132-3p mimic vs miR-132-3p mimic+GLRX	3	Yes					Tukey	0.0103
7*A*	6	Yes	One-way ANOVA	*F*_(3,20)_ = 65.02	<0.0001	Treatment		
7*A*, saline vs MPTP	6	Yes					Tukey	<0.0001
7*A*, MPTP+ antagomir NC vs MPTP+miR-132-3p antagomir	6	Yes					Tukey	0.001
7*B*	6	Yes	One-way ANOVA	*F*_(3,20)_ = 35.37	<0.0001	Treatment		
7*B*, saline vs MPTP	6	Yes					Tukey	<0.0001
7*B*, MPTP+ antagomir NC vs MPTP+miR-132-3p antagomir	6	Yes					Tukey	0.001

### Western blotting

For collection of protein samples, RIPA lysis buffer (Beyotime Biotech) was used to treat cells or tissues. Following determination of protein concentration with a BCA kit, the proteins in the corresponding volume were mixed with loading buffer (Beyotime) and subjected to denaturation in a boiling-water bath for 3 min. Electrophoresis was embarked at 80 V for 30 min, and then for 1–2 h at 120 V when bromphenol blue reached the separation gel. The proteins were transferred onto membranes at 300 mA for 60 min in an ice bath. Then, the membranes were rinsed 1–2 min with washing solution and inactivated in the blocking solution at room temperature for 60 min, or sealed overnight at 4°C. Following incubation with the primary antibodies against β-actin (ab8226, 1  μg/ml) and GLRX (ab45953, 1:250; Abcam) at room temperature in a shaking table for 1 h, the membranes were washed with the washing solution for 3 × 10 min and incubated with the secondary antibody for 1 h at room temperature. The membranes were washed thrice for 10 min and exposed to developing liquid for color development. Then, the membranes were observed in chemiluminecence imaging analysis system (Gel Doc XR, Bio-Rad).

### ELISA

The ELISA kit (R&D Systems) was adopted to determine the contents of TNF-α, IL-6, and IL-1β in the cell supernatant. All operations were performed in accordance with the instructions of the ELISA kit.

### Coculture of BV-2 and SH-SY5Y cells

The effect of microglial activation on SH-SY5Y cells was studied by coculture of BV-2 cell supernatant and SH-SY5Y cells. The supernatant of BV-2 cells in each group was collected and filtered with a 0.45-μm filter. SH-SY5Y cells were seeded onto the six-well plates for cell culture till the density of SH-SY5Y cells reached 70%. The supernatant of BV-2 cells and DMEM/F12 containing 10% FBS were mixed at a ratio of 1:1, and the mixture was co-cultured with SH-SY5Y cells for 24 h.

### CCK-8 assay

The SH-SY5Y cells were seeded onto 96-well plates, and cells in each well received 100-μl prediluted cell suspension (1 × 10^5^ cells/ml). Twenty-four hours later, SH-SY5Y cells were grown in conditioned medium of BV-2 cells for 24 h. The experiment was designed with three replicates. Ten microliters of CCK-8 solution (Dojindo) was added to each well for 2 h of incubation. The optical density (OD) at 450-nm wavelength was assessed.

### Flow cytometry

After SH-SY5Y cells (10^5^ cells/ml) were incubated with conditioned medium of BV-2 cells for 24 h, 3-ml cell suspension from each sample was transferred into a 10-ml centrifuge tube for 5 min of centrifugation at 500 rpm. After removal of culture medium, cells were washed with PBS and centrifuged at 500 rpm for 5 min. The supernatant was discarded. Then, cells were resuspended in 100 μl of binding buffer, and then gently mixed with 5-μl Annexin V-fluorescein isothiocyanate (FITC) and 5 μl PI for incubation for 15 min in the dark. The fluorescence of FITC and PI was examined by flow cytometry, and the apoptosis rate was analyzed.

### RNA immunoprecipitation (RIP)

Following wash twice with precooled PBS, cells were centrifuged at 1500 rpm for 5 min and lysed with equivoluminal RIP lysis buffer. The magnetic beads were resuspended in 100-μl RIP Wash buffer followed by 30 min of incubation with 5-μg antibody against Ago2 (ab186733, 1:30, Abcam) or IgG (ab172730, negative control) at room temperature. Cells in the centrifuge tube were placed on a magnetic separation rack to discard the supernatant. Following incubation with 500- μl RIP Wash buffer for vortex oscillation twice, cells were given 500  μl of RIP Wash buffer for vortex oscillation and placed on ice. The magnetic bead tube was transferred to the magnetic separation rack, and the supernatant was removed. After that, cells in each tube received 900  μl of RIP immunoprecipitation buffer. Cell lysates were centrifuged at 14,000 rpm at 4°C for 10 min, and 100  μl of supernatant was pipetted into the magnetic bead-antibody complex for incubation overnight at 4°C. The complex processed centrifugation with supernatant removed. Then, the centrifuge tube received 500  μl of RIP Wash buffer for vortex oscillation and cell supernatant was abandoned before the sediments were washed for six times. The magnetic bead-antibody complex was resuspended in 150 μl of Proteinase K buffer and incubated at 55°C for 30 min. Then, the samples were put in the magnetic separation rack to remove the supernatant. The gene expression was analyzed by qRT-PCR after RNA extraction.

### Dual-luciferase reporter assay

The binding site of *miR-132-3p* and GLRX was predicted by the online prediction software StarBase (http://starbase.sysu.edu.cn/). The mutated type and wild-type sequences in the binding sites were designed and cloned into pGL3-Promoter luciferase plasmid (Promega), namely mut-GLRX and wt-GLRX. Then, mut-GLRX or wt-GLRX was cotransfected with *miR-132-3p* mimic or *miR-132-3p* inhibitor, respectively, into HEK-239T cells or pRL-TK (Promega). After that, *Renilla* luciferase activity and Firefly luciferase activity were determined by dual-luciferase reporter gene assay kit (Promega). *Renilla* luciferase activity was deemed as the internal control, and the ratio between the activities of Firefly luciferase and *Renilla* luciferase was calculated as the relative activity.

### PD mouse model

Six-month-old male C57BL/6J mice (*n* = 24) were purchased from the Shanghai SLAC Laboratory Animal Co, Ltd. All animal handling and experimental procedures were approved by the Animal Care and Use Committee of Hunan Provincial People’s Hospital (No. 202004). Mice were allowed food and water *ad libitum* and housed in rooms maintained at 24 ± 1°C and 60–80% humidity using a 12-h dark cycle. The following experiments were conducted after one week of feeding. The study consisted of four groups of six mice each (random grouping by an Excel random number generator): the 1-methyl-4-phenyl-1,2,3,6-tetrahydropyridine (MPTP), saline, *miR-132-3p* antagomir and antagomir NC groups. Mice in the MPTP group were intraperitoneally injected with 30 mg/kg MPTP (Sigma-Aldrich) every day for five consecutive days (Hu et al., 2019), and the mice of the Saline group were intraperitoneally injected with the same amount of Saline every day for five consecutive days. Mice in the MPTP*+miR-132-3p* antagomir group or MPTP+antagomir NC group were injected with MPTP the next day after stereotactic injection of *miR-132-3p* antagomir or antagomir NC (20 nm, total volume of 5 μl, GenePharma) into targeted brain areas of mice.

### Stereotactic injection

After anesthesia of mice with ketamine (100 mg/kg) and xylazine (10 mg/kg) by intraperitoneal injection, the head of mouse was fixed to expose the skull. The intracerebral injection was performed on the following coordinates: −2.8 mm anteroposterior, −1.2 mm mediolateral, and −4.3 mm dorsoventral. Five microliters of *miR-132-3p* antagomir suspension or antagomir NC suspension was injected into SNc by using a 10-μl stereotactic catheter (1 μl/5 min). The needle remained in place for 5 min after complete injection then slowly removed. The mice were placed on a pad until recovery from the anesthesia. The healthy conditions of mice were monitored on the following 5 d, during which mice were subjected to acesodyne and local disinfection. The activity of mice after injection was recorded. No mouse was died during the whole experiments.

### Behavioral tests

One week after establishment of PD mouse models, the behavioral tests were commenced. Motor coordination ability of experimental animals was investigated with the rotarod test. Before the experiments, animals were placed on rotating lanes for 5 min. Then, mice were trained for 2 min at a fixed speed of 4 rpm. After training, the rotational speed was accelerated uniformly from 4 rpm to 40 rpm within 60 s. The time of mice falling off the rotating rod was recorded. The open field test was conducted to evaluate the autonomous and exploratory behaviors of experimental animals in novel environments. Mice were individually placed into the center of an open field box (38 × 38 cm) in a noise and light-controlled room. The spontaneous locomotor activities (central-area distance and whole-area distance) of each mouse were recorded and analyzed. The relative time of mouse falling from the rotating rod and the relative distance of mouse staying in the open field were recorded. Relative time and relative distance are the ratio of time or distance of experimental mouse/control mouse.

### Brain tissue collection

Approximately 24 h after behavior test, ketamine (100 mg/kg) and xylaafine (10 mg/kg) were given to mouse for anesthesia through intraperitoneal injection. The heart was exposed and mouse (*n* = 6) in each group was perfused with 200 ml of normal saline through ventriculus sinister. The skull was opened and the brain tissues were collected and isolated. Part of the brain tissues was stored at −80°C refrigerator for qRT-PCR and the rest brain tissues were fixed in 4% triformol for 48 h for fluorescence *in situ* hybridization (FISH), immunofluorescence and immunohistochemistry.

### FISH

The 4-μm paraffin sections were de-paraffinized with xylene and gradient alcohol (xylene soak for 10 min, refresh xylene for another 10 min, 50% xylene soaking for 10 min, absolute ethanol for 5 min, refresh absolute ethanol for another 5 min, 95% ethanol for 5 min, 90% ethanol for 5 min, 80% ethanol for 5 min, and 70% ethanol for 5 min) before PBS wash. Sections were digested with 37°C protease K for 15 min, washed with PBS for 2 × 5 min, prehybridized at 55°C constant temperature, and incubated with digoxigenin-labeled (Exiqon) *miR-132-3p* probes overnight at incubator with 55°C constant temperature. Then, sections were subsequently washed with 5 × SSC buffer, 1 × SSC, and 0.2 × SSC buffer for 2 × 5 min at 55°C, followed by 5 min of wash with 0.2 × SSC buffer at room temperature, 10 min of inactivation with 0.3% hydrogen peroxide-methanol solution, and 3 × 5 min of PBS wash. After that, sections underwent three times of incubation each for 1 h: first blocked with normal serum blocking buffer at room temperature, second probed with mouse anti-DIG at room temperature, and then incubated with polymer anti-mouse. After each incubation, sections were washed three times with PBS for 5 min. Sections were stained with DAB for 5–10 min and washed with tap water for 10 min, before 2 min of hematoxylin counterstaining, hydrochloric ethanol differentiation and 10 min of tap water wash. These sections were sealed with neutral balata for observation under a microscope after dehydration and permeabilization. The ratio of positive cell numbers to total number of cells was calculated. DAPI was used for staining of cell nucleus and Iba1 was used to labeled microglial cells.

### Immunofluorescence

Sections were incubated for 60 min at 60°C, dewaxed with xylene and washed with distilled water. Following antigen retrieval with 0.01 mol/l sodium citrate, sections were subjected to 10 min of incubation with 3% H_2_O_2_. Then, sections were washed with PBS for 3 × 5 min, and inactivated with 5% normal goat serum for 30 min at room temperature. Sections were cultured with the primary antibody against tyrosine hydroxylase (ab137869, 1:200, Abcam), Iba1 (ab178846, 1:500, Abcam) or GLRX (ab45953, 1  μg/ml, Abcam) overnight at 4°C, while sections in negative control group contains corresponding antigens and were incubated with PBS. After that, sections were washed three times with PBS and incubated with FITC-labeled secondary antibody (ab6785, 1:1000, Abcam) at room temperature for 1 h. Then, the secondary antibody was removed, and cells were subjected to 5 min of staining with DAPI and 3 × 5 min of PBS wash. Before pictures were captured by fluorescence microscope, sections were given glycerophosphoric acid for sealing. StereoInvestigator (MBF Bioscience) was used for stereological analysis on the total number of TH positive neurons and Iba1 positive cells for every sixth coronal section through the midbrain. After the the SN pars compacta with a 4× objective was delineated, cells were counted under × 60 magnification using ImageJ and following parameters: 8- μm height of an optical dissector, 50 × 50 μm counting frame, 100 × 100 μm area of a grid. The error coefficient of <0.10 was acceptable. All sections were quantified in a blinded manner.

### Immunohistochemistry

Following 60 min of bake, sections were dewaxed by xylene and washed with distilled water. Before 30 min of inactivation with normal goat serum at room temperature, sections underwent the following steps: antigen retrieval with 0.01 mol/l sodium citrate, 10 min of reaction with 3% H_2_O_2_ and three times of PBS wash for 5 min. The sections were inactivated with 5% normal goat serum for 30 min at room temperature. After that, sections were incubated with antibody against GLRX (ab45953, 1  μg/ml, Abcam) overnight at 4°C, while sections in negative control group contains corresponding antigens and were incubated with PBS. Sections were then subjected to three times of PBS wash and 1 h of incubation with secondary antibody (ab6785, 1:1000, Abcam). DAB was used for color development, and sections were given three times of PBS wash to terminate the color reaction (1–3 min). The nucleus was stained with hematoxylin for 3 min, and sections were dehydrated, permeabilized, and sealed. The percentage of positive cells was counted. Images were analyzed using ImageJ software (version 1.46, National Institutes of Health).

### Statistical analysis

Experiments and statistical analysis were performed by different personnel. Statistical analysis was conducted using GraphPad Prism 7 software, and data are displayed as the mean ± SD. The normal distribution of data was detected by Kolmogorov–Smirnov test, D’Agostino, Pearson omnibus normality test, or Shapiro–Wilk normality test. All data were complied with normal distribution. The *t* test was employed for comparisons between two groups. The one-way ANOVA was adopted followed by Tukey’s multiple comparison tests for comparisons among multiple groups. *P* values of significance were those <0.05 (Tables 2, 3, 4).

## Results

### Highly expressed *miR-132-3p* and lowly expressed GLRX in midbrain tissues of patients with PD

**Figure 1.**
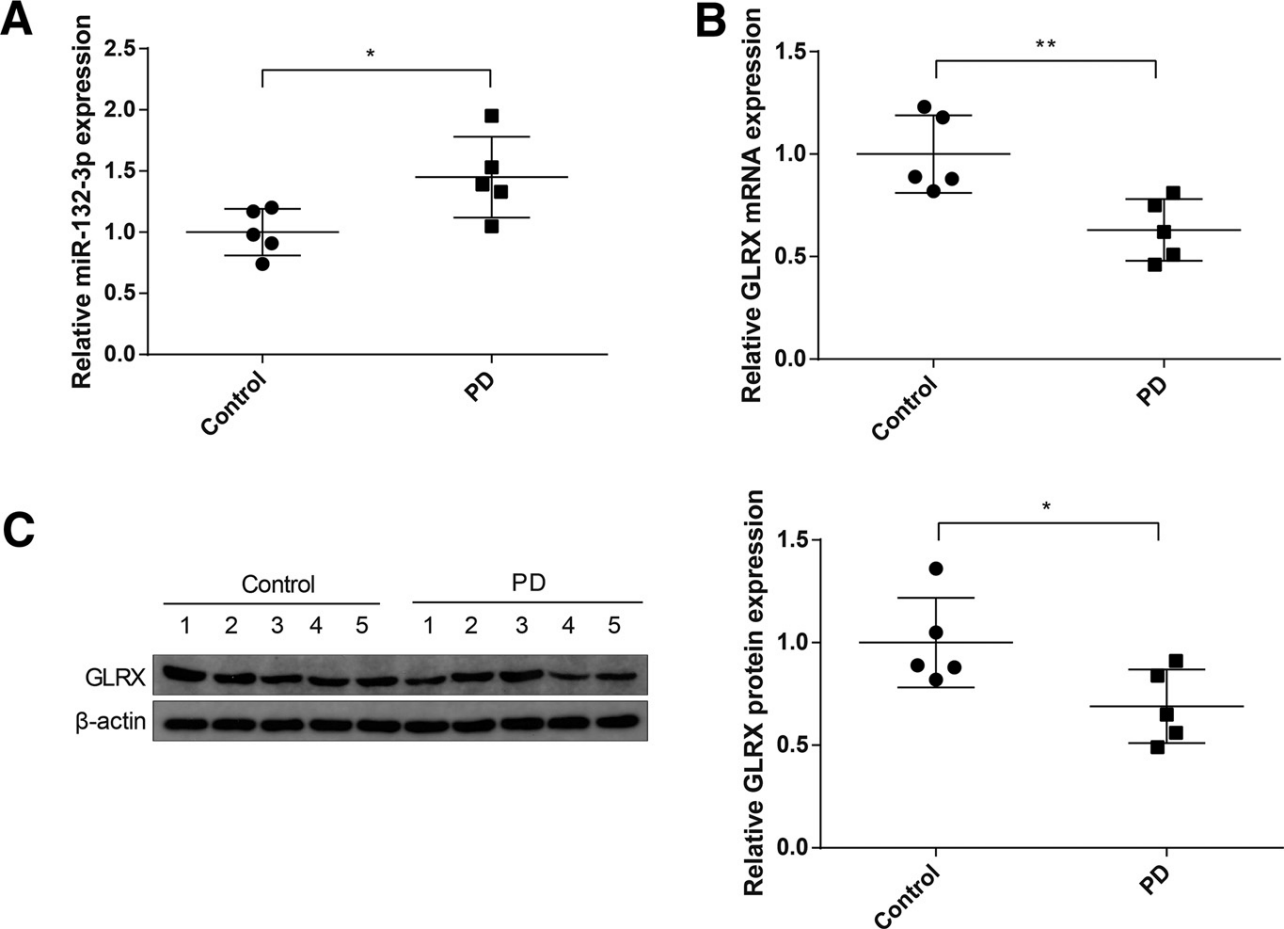
Expressions of *miR-132-3p* and GLRX in the midbrain tissues of patients with PD. The expression of *miR-132-3p* in the midbrain tissues of five PD patients and five age-matched controls was detected by qRT-PCR (***A***), and the mRNA and protein expressions of GLRX were measured by qRT-PCR (***B***), and Western blotting (***C***); *N* (number of participants) = 5, *p < 0.05, **p < 0.01, Error bars, standard deviation (SD).

The expressions of *miR-132-3p* and GLRX in midbrain tissues of patients with PD and in healthy controls were determined by qRT-PCR and Western blotting. The results of qRT-PCR manifested compared with control group, *miR-132-3p* in midbrain tissues of patients with PD was increased by 1.45 ± 0.33-fold (*p* < 0.05; Fig. 1*A*). Furthermore, analyses of qRT-PCR and Western blotting exhibited that the mRNA and protein expressions of GLRX in the tissues of patients with PD were decreased to respectively 0.63 ± 0.15-fold and 0.69 ± 0.18-fold (*p *< 0.05, vs the control group; Fig. 1*B*,*C*). These finding indicated that *miR-132-3p* and GLRX may be implicated in the progression of PD.

### Knock-down of *miR-132-3p* inhibits LPS-induced inflammatory response in BV-2 cells

**Figure 2.**
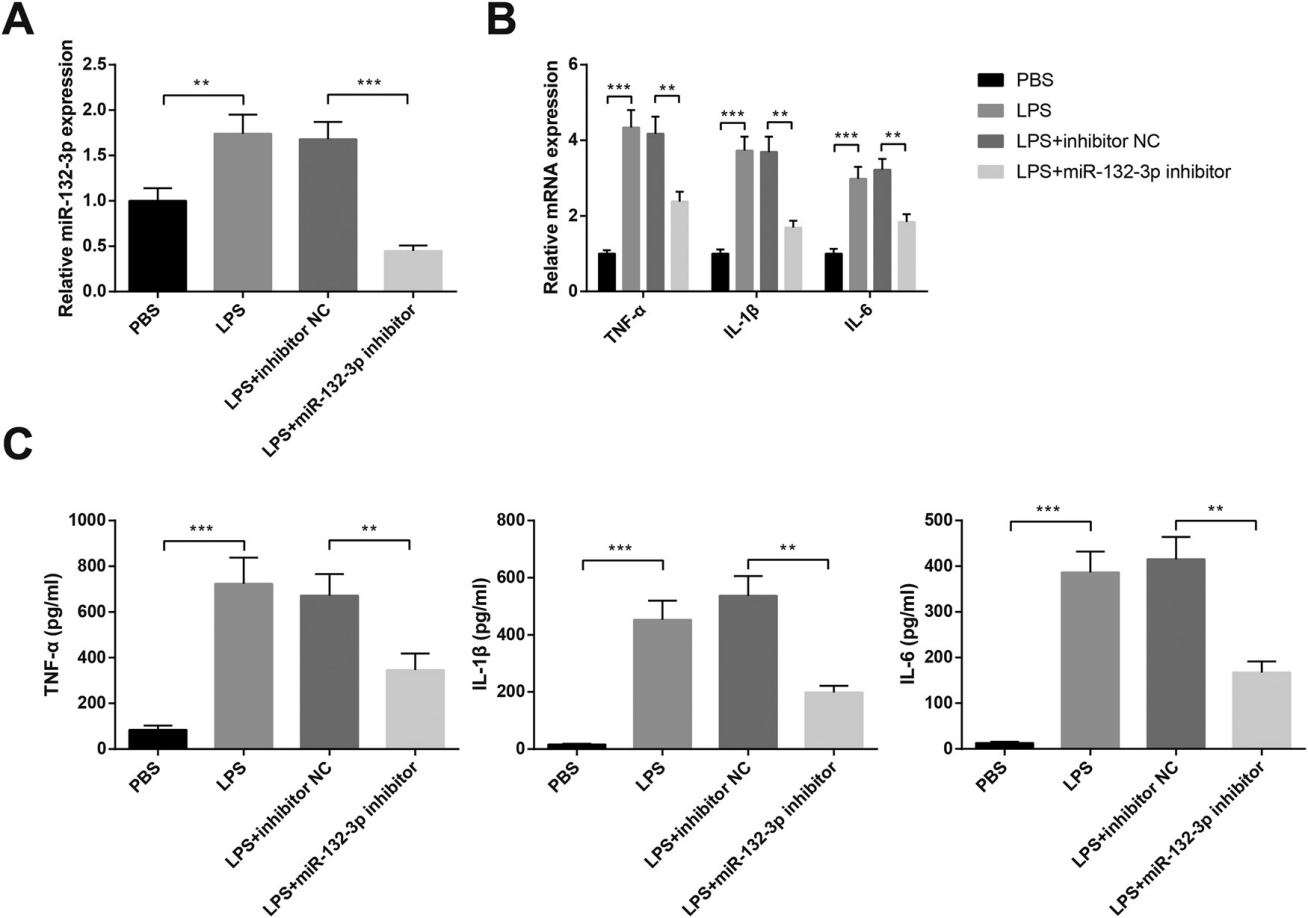
LPS-induced inflammatory response in BV-2 microglial cells can be attenuated by *miR-132-3p* knock-down. Following transfection with *miR-132-3p* inhibitor or inhibitor NC, BV-2 cells were treated with 0.1 μg/ml LPS or PBS for 24 h. qRT-PCR was used to detect the expression of *miR-132-3p* in BV-2 cells (***A***). The mRNA expressions of inflammatory cytokines TNF-α, IL-1β, and IL-6 were analyzed by qRT-PCR (***B***), and the contents of TNF-α, IL-1β, and IL-6 in the supernatant of BV-2 cells were determined by ELISA (***C***); *N* (number of independent cell culture preparations) = 3, **p < 0.01, ***p < 0.001, Error bars, standard deviation (SD).

The BV-2 cells were transfected with *miR-132-3p* inhibitor or inhibitor NC before 0.1 μg/ml LPS or PBS treatment to explore the effect of *miR-132-3p* on LPS-induced inflammatory response in BV-2 cells. Results of qRT-PCR presented that LPS treatment elevated *miR-132-3p* expression in BV-2 cells by 1.74 ± 0.21-fold (*p *<* *0.01, vs the PBS group), while transfection with *miR-132-3p* inhibitor suppressed the level of *miR-132-3p* (*p* < 0.001, LPS + miR-132-3p inhibitor vs the LPS+inhibitor NC group: 0.45 ± 0.06-fold vs 1.68 ± 0.19-fold; Fig. 2*A*). Additionally, the enhanced expressions of inflammatory cytokines IL-6 (2.98 ± 0.32-fold), TNF-α (4.34 ± 0.46-fold) and IL-1β (3.73 ± 0.37-fold) in BV-2 cells were occurred in response to LPS induction (*p *<* *0.001, vs the PBS group), whereas these levels were somewhat attenuated in cells transfected with *miR-132-3p* inhibitor (*p* < 0.01, LPS+miR-132-3p inhibitor vs the LPS+inhibitor NC group: TNF-α: 2.38 ± 0.26-fold vs 4.18 ± 0.45-fold; IL-1β: 1.69 ± 0.18-fold vs 3.68 ± 0.41-fold; IL-6: 1.84 ± 0.21-fold vs 3.22 ± 0.29-fold; Fig. 2*B*). The contents of inflammatory cytokines in the supernatant of BV-2 cells were determined by ELISA, and the results showed that the rises in contents of IL-1β (453.29 ± 66.47 vs 16.74 ± 2.58 pg/ml), IL-6 (386.47 ± 45.79 vs 13.27 ± 2.18 pg/ml) and TNF-α (734.48 ± 114.37 vs 84.56 ± 18.24 pg/ml) in the LPS group (*p *<* *0.001, vs the PBS group). Compared with LPS+inhibitor NC group, contents of IL-1β (198.37 ± 23.51 vs 537.28 ± 68.34 pg/ml), IL-6 (167.28 ± 24.35 vs 415.27 ± 48.61 pg/ml), and TNF-α (346.57 ± 72.44 vs 672.35 ± 94.27 pg/ml) in the LPS+*miR-132-3p* inhibitor group were suppressed (*p* < 0.01; Fig. 2*C*). The above findings suggested that suppression on *miR-132-3p* may repress LPS-induced inflammatory response in BV-2 microglial cells.

### *miR-132-3p* overexpression enhances inflammatory response in BV-2 cells

**Figure 3.**
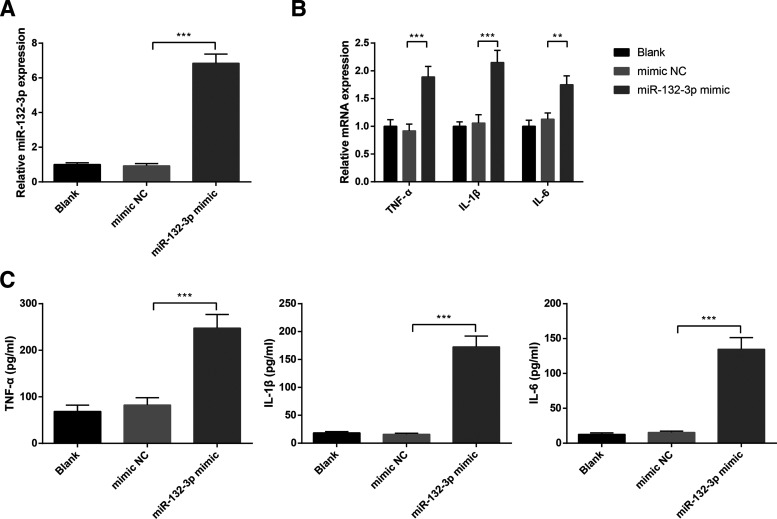
Overexpression of *miR-132-3p* promotes the release of proinflammatory cytokines in BV-2 microglial cells. After BV-2 cells transfected with *miR-132-3p* mimic or mimic NC, the mRNA expression of *miR-132-3p* in BV-2 cells (***A***) and the mRNA expressions of inflammatory cytokines TNF-α, IL-1β, and IL-6 in BV-2 cells were examined by qRT-PCR (***B***). Then, ELISA was utilized to assess the contents of TNF-α, IL-1β, and IL-6 in the supernatant of BV-2 cells (***C***); *N* (number of independent cell culture preparations) = 3, **p < 0.01, ***p < 0.001, Error bars, standard deviation (SD).

Based on the previous experimental results, downregulation of *miR-132-3p* can inhibit LPS-induced activation and inflammation of microglial cells. However, whether upregulation of *miR-132-3p* can induce microglial activation and inflammation is still unknown. Toward this end, BV-2 cells were transfected with *miR-132-3p* mimic or mimic NC. We found that overexpression of *miR-132-3p* in miR-132-3p mimic group had increased the levels of *miR-132-3p* by 6.84 ± 0.53-fold (*p* < 0.001; Fig. 3*A*). Additionally, miR-132-3p mimic group had elevated mRNA expressions of TNF-α (1.89 ± 0.19-fold vs 0.92 ± 0.12-fold), IL-1β (2.15 ± 0.22-fold vs 1.06 ± 0.15-fold), IL-6 (1.75 ± 0.16-fold vs 1.13 ± 0.11-fold) when compared with mimic NC group (*p* < 0.01; Fig. 3*B*). ELISA showed that TNF-α (247.63 ± 29.14 vs 82.78 ± 15.69 pg/ml), IL-1β (172.59 ± 19.37 vs 15.94 ± 1.83 pg/ml), IL-6 (134.76 ± 16.72 vs 15.36 ± 1.87 pg/ml) in the supernatant of BV-2 cells was also elevated in miR-132-3p mimic group in contrast to mimic NC group (*p* < 0.001; Fig. 3*C*). The results demonstrated that overexpression of *miR-132-3p* may induce the activation and inflammatory response of microglial cells.

### Activated microglial cells by *miR-132-3p* cause neuronal injury

**Figure 4.**
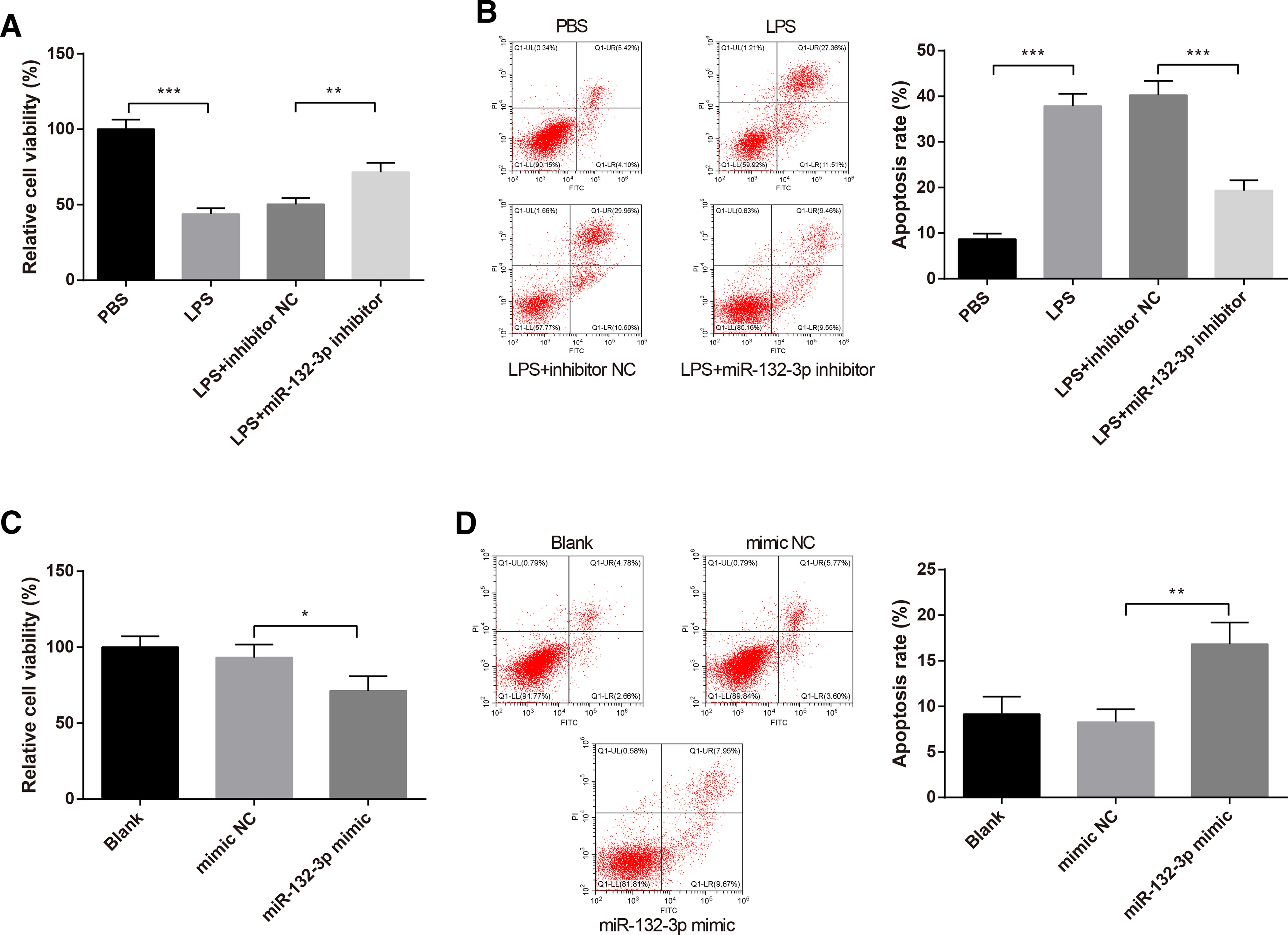
Effect of *miR-132-3p* induced microglial activation on neuronal injury. The SH-SY5Y cells were cultured in the conditioned medium of BV-2 cells that were transfected with inhibitor NC or *miR-132-3p* inhibitor and stimulated with LPS. Then, CCK-8 assay was used to detect the viability of SH-SY5Y cells (***A***) and flow cytometry to determine the apoptotic rate (***B***). Additionally, SH-SY5Y cells were cultured in the conditioned medium of BV-2 cells that transfected with *miR-132-3p* mimic or mimic NC. The viability of SH-SY5Y cells was assessed by CCK-8 assay (***C***) and the apoptotic rate of SH-SY5Y cells was measured by flow cytometry (***D***); *N* (number of independent cell culture preparations) = 3, *p < 0.05, **p < 0.01, ***p < 0.001, Error bars, standard deviation (SD).

To investigate the role of microglial activation in neuronal injury, SH-SY5Y cells were cultured in conditioned medium of BV-2 cells that were transfected with inhibitor NC or *miR-132-3p* inhibitor and stimulated with LPS. The findings of CCK-8 assay addressed that the viability of SH-SY5Y cells in the LPS group was decreased to 43.86 ± 3.86% compared with PBS group (*p *<* *0.001), while LPS+*miR-132-3p* inhibitor group possessed higher cell viability than that in the LPS+inhibitor NC group (*p* < 0.01, 71.61 ± 6.23% vs 50.27 ± 4.14%; Fig. 4*A*). Flow cytometry assessed that LPS stimulation heightened the apoptotic rate of SH-SY5Y cells (*p* < 0.001, 37.84 ± 2.73% vs 8.67 ± 1.23%; Fig. 4*B*), but treatment with *miR-132-3p* inhibitor lowered cell apoptotic rate (*p* < 0.001, 19.34 ± 2.27% vs 40.25 ± 3.16%; Fig. 4*B*).

Meanwhile, the effect of *miR-132-3p* overexpression on neuronal injury was explored. Accordingly, SH-SY5Y cells were cultured in conditioned medium, in which BV-2 cells were transfected with mimic NC or *miR-132-3p* mimic. We discovered that upregulation of *miR-132-3p* reinforced cell apoptotic rate (16.83 ± 2.37% vs 8.25 ± 1.43%) and diminished the viability of SH-SY5Y cells (71.35 ± 9.61% vs 93.15 ± 8.63%; Fig. 4*C*,*D*, *p *<* *0.05). Collectively, the activated microglial cells by *miR-132-3p* may lead to neuronal injury.

### *MiR-132-3p* is an upstream regulatory factor of GLRX

**Figure 5.**
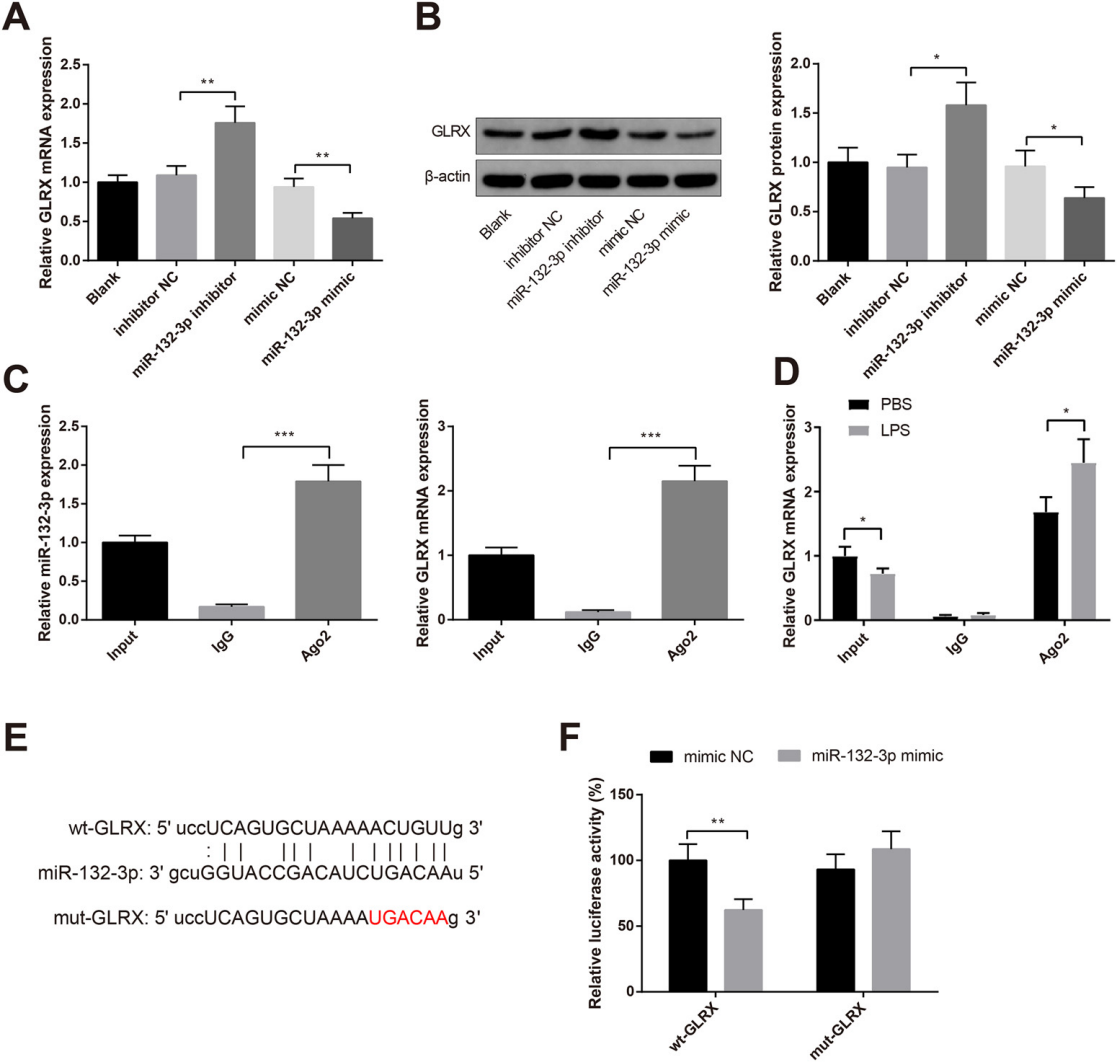
*MiR-132-3p* negatively mediates GLRX. qRT-PCR (***A***) and Western blotting (***B***) were used to detect the mRNA and protein expressions of GLRX after *miR-132-3p* knock-down or overexpression in BV-2 cells. RIP experiment was applied to verify the binding of *miR-132-3p* to GLRX mRNA (***C***). After LPS or PBS treatment, RIP was applied to detect the GLRX mRNA expression in Ago2 complex (***D***). The binding site of *miR-132-3p* to the 3′-UTR of GLRX mRNA was predicted by StarBase (***E***). Dual-luciferase reporter assay was utilized to verify the binding relationship between *miR-132-3p* and GLRX (***F***); *N* (number of independent cell culture preparations) = 3, *p < 0.05, **p < 0.01, ***p < 0.001, Error bars, standard deviation (SD).

After knock-down or overexpression of *miR-132-3p* in BV-2 cells, the mRNA and protein expressions of GLRX were evaluated by qRT-PCR and Western blotting. We found that enhanced GLRX expression in miR-132-3p inhibitor group when compared with inhibitor NC group (*p *<* *0.05, mRNA: 1.76 ± 0.21-fold vs 1.09 ± 0.12-fold; protein: 1.58 ± 0.23-fold vs 0.95 ± 0.13-fold; Fig. 5*A*,*B*), while miR-132-3p mimic group had repressed level of GLRX compared with mimic NC group (*p *<* *0.05, mRNA: 0.54 ± 0.07-fold vs 0.94 ± 0.11-fold; protein: 0.64 ± 0.11-fold vs 0.96 ± 0.16-fold; Fig. 5*A*,*B*). Subsequently, the RIP experiment was applied to verify the relationship of *miR-132-3p* to GLRX mRNA, and the results manifested that compared with IgG antibody group, substantial GLRX mRNA can be recruited in Ago2 complex (2.15 ± 0.24-fold vs 0.12 ± 0.03-fold; Fig. 5*C*, *p* < 0.001). After LPS treatment, increased GLRX mRNA was recruited in Ago2 complex (2.45 ± 0.36-fold vs 1.68 ± 0.23-fold; Fig. 5*D*, *p* < 0.05). Considering the directing binding of miR-132-3p with GLRX mRNA cannot be proved by RIP as the regulation of other miRNAs or target genes cannot be excluded, we then applied dual luciferase reporter gene assay to verify the binding. The binding site of *miR-132-3p* to the 3′-UTR on GLRX mRNA was predicted by StarBase (Fig. 5*E*). The above result was verified by dual-luciferase reporter assay, which displayed that HEK293T cells cotransfected with wt-GLRX and *miR-132-3p* mimic had decreased luciferase activity by 62.33 ± 8.17% than cells cotransfected with wt-GLRX and mimic NC (*p *<* *0.01). However, the relative luciferase activity in cells cotransfected with mut-GLRX and *miR-132-3p* mimic was not statistically different from cells cotransfected with mut-GLRX and mimic NC (*p* > 0.05, 108.67 ± 13.52% vs 93.16 ± 11.47%; Fig. 5*F*). The above results indicated that *miR-132-3p* may target GLRX and negatively regulate expression of GLRX.

### GLRX mitigates neuronal injury and inhibits activation of microglial cells induced by *miR-132-3p*

**Figure 6.**
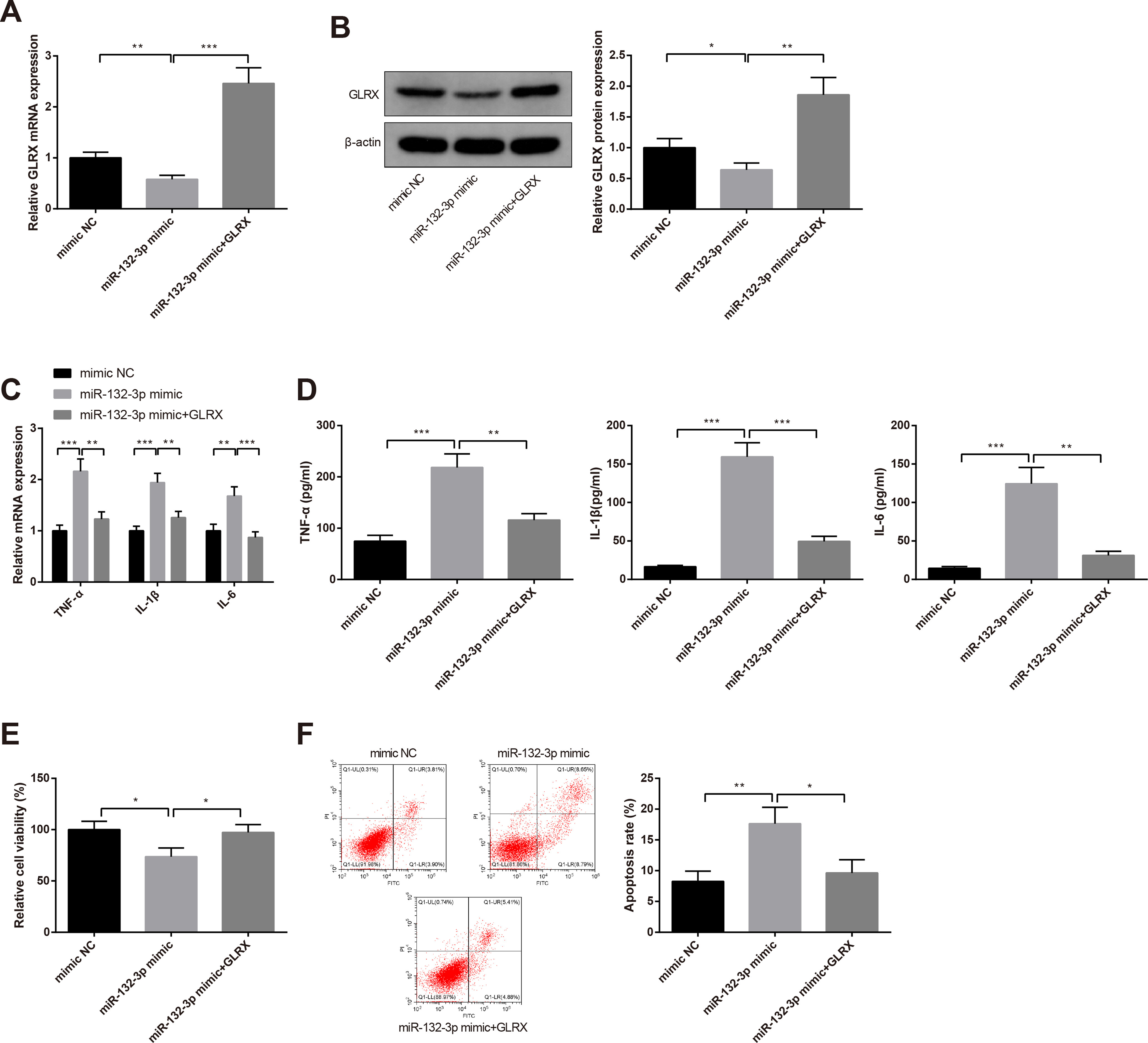
GLRX reverses microglial activation and neuronal injury induced by *miR-132-3p*. The BV-2 cells were transfected *miR-132-3p* mimic or cotransfected *miR-132-3p* mimic and GLRX overexpressing plasmid. Then, qRT-PCR (***A***) and Western blotting (***B***) were used to detect the mRNA and protein expressions of GLRX in BV-2 cells. The mRNA expressions of inflammatory cytokines TNF-α, IL-1β, and IL-6 in BV-2 cells were examined by qRT-PCR (***C***). Then, ELISA was utilized to assess the contents of TNF-α, IL-1β, and IL-6 in the supernatant of BV-2 cells (***D***). The SH-SY5Y cells were cultured in conditioned medium, in which BV-2 cells were transfected with *miR-132-3p* mimic or cotransfected *miR-132-3p* mimic and GLRX overexpressing plasmid. Then, CCK-8 assay was used to detect the viability of SH-SY5Y cells (***E***) and flow cytometry to determine the apoptotic rate (***F***); *N* (number of independent cell culture preparations) = 3, *p < 0.05, **p < 0.01, ***p < 0.001, Error bars, standard deviation (SD).

To explore whether *miR-132-3p* promotes microglial inflammation and neuronal injury through mediating GLRX, BV-2 cells were transfected *miR-132-3p* mimic or cotransfected *miR-132-3p* mimic and plasmid overexpressing GLRX. Results of qRT-PCR and Western blotting highlighted that transfection with *miR-132-3p* mimic lead to suppressed mRNA and protein expression levels of GLRX by 0.58 ± 0.08-fold and 0.64 ± 0.11-fold, whereas the coeffect of *miR-132-3p* mimic and plasmid overexpressing GLRX increased the expression of GLRX compared with transfection with *miR-132-3p* mimic alone (*p *<* *0.05, mRNA: 2.46 ± 0.31-fold vs 0.58 ± 0.08-fold; protein: 1.85 ± 0.28-fold vs 0.64 ± 0.11-fold; Fig. 6*A*,*B*). Furthermore, the detections on mRNA expressions and contents of inflammatory cytokines revealed that cells in the *miR-132-3p* mimic+GLRX group had lower mRNA expression levels and contents of TNF-α (1.23 ± 0.14-fold vs 2.16 ± 0.22-fold), IL-6 (0.87 ± 0.11-fold vs 1.68 ± 0.18), and IL-1β (1.26 ± 0.12-fold vs 1.94 ± 0.18-fold) than in the *miR-132-3p* mimic group (*p* < 0.01; Fig. 6*C*). ELISA detection showed that the contents of TNF-α (115.86 ± 12.43 vs 218.37 ± 26.49 pg/ml), IL-1β (49.37 ± 6.68 vs 159.38 ± 18.32 pg/ml), IL-6 (31.27 ± 5.39 vs 124.38 ± 21.15 pg/ml) in the supernatant of BV-2 cells were suppressed in miR-132-3p mimic+GLRX group when compared with miR-132-3p mimic group (*p* < 0.01; Fig. 6*D*). Then, SH-SY5Y cells were grown in the conditioned medium of BV-2 cells. The findings of CCK-8 assay and flow cytometry described the increase in cell viability (97.39 ± 7.65% vs 73.62 ± 8.51%; Fig. 6*E*, *p* < 0.05) and the decrease in cell apoptotic rate (9.66 ± 2.16% vs 17.59 ± 2.67%; Fig. 6*F*, *p* < 0.01) in the *miR-132-3p* mimic+GLRX group compared with the *miR-132-3p* mimic group. These data suggested that overexpression of GLRX may reverse the effect of *miR-132-3p* upregulation on microglial activation and neuronal injury.

### Suppression of *miR-132-3p* alleviates MPTP-induced neuroinflammation and dopaminergic neurodegeneration in PD mouse models

**Figure 7.**
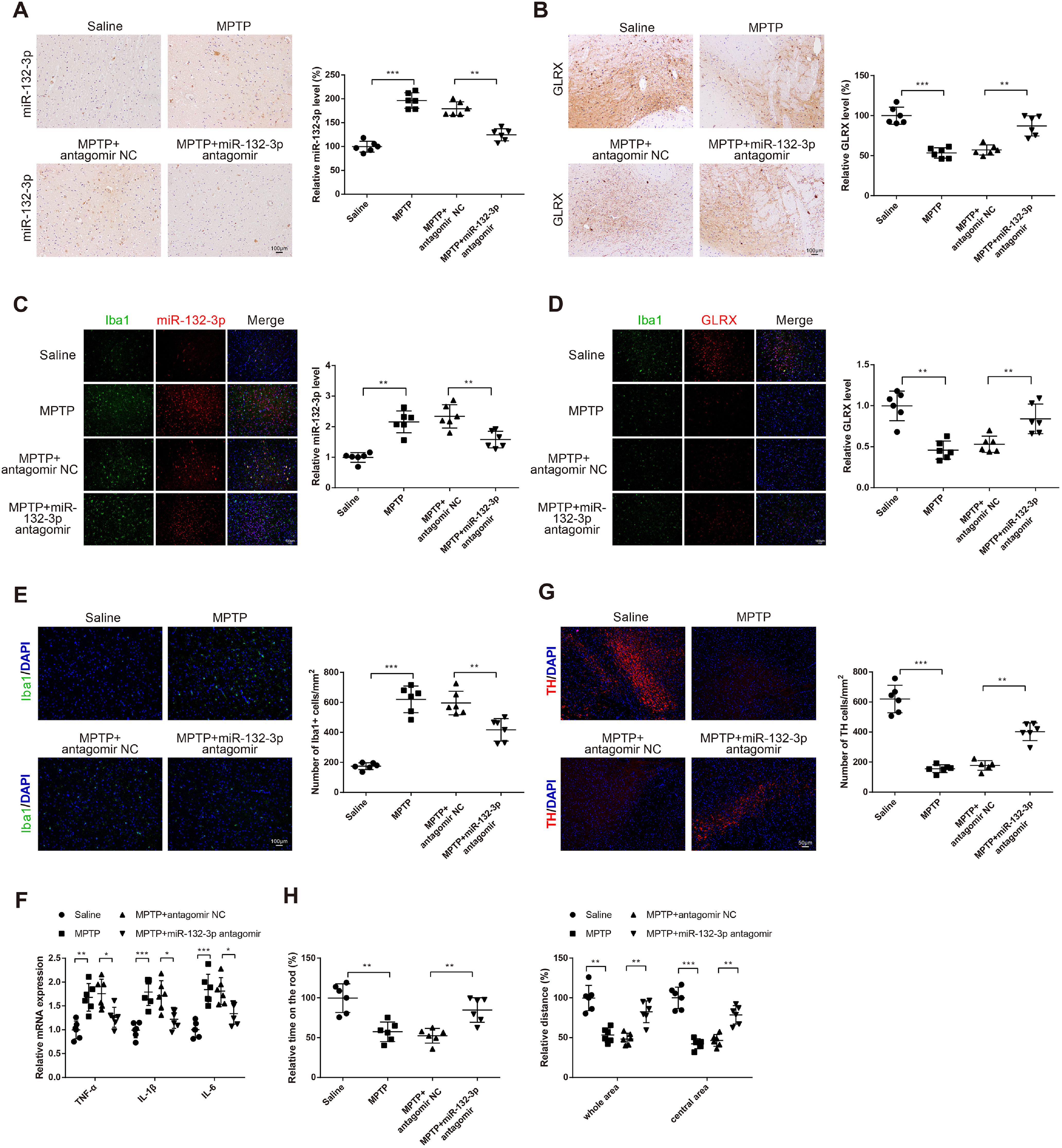
Knock-down of *miR-132-3p* ameliorates the neuroinflammation and dopaminergic neuron degeneration of PD mouse. Mice were intraperitoneally injected with 30 mg/kg MPTP to establish PD mouse models. Then, mouse models of PD were given stereotactic injection of *miR-132-3p* antagomir or antagomir NC. FISH was used to examine the expression of *miR-132-3p* in SNc of mice (***A***). The expression of GLRX in the SNc of mice was measured by immunohistochemistry (***B***). FISH was applied to detect the expression of miR-132-3p in microglial cells (***C***). Immunofluorescence was applied to detect the expression of GLRX in microglial cells (***D***); Immunofluorescence of Iba1 was applied to detect the activation of microglial cells (***E***), qRT-PCR to detect the mRNA expressions of TNF-α, IL-1β, and IL-6 in brain tissues of mouse (***F***), and immunofluorescence of tyrosine hydroxylase to detect the loss of dopaminergic neurons in the SNc of mice (***G***). The motor ability of mice was assessed after rotarod test and open field test (***H***); *N* (number of animals) = 6, *p < 0.05, **p < 0.01, ***p < 0.001, Error bars, standard deviation (SD).

Mice were subjected to stereotactic injection of *miR-132-3p* antagomir and given MPTP by intraperitoneal injection to probe the role of *miR-132-3p* in neuroinflammation and dopaminergic neuron degeneration of MPTP-induced PD mouse. The results of FISH presented that injection with MPTP elevated *miR-132-3p* expression in SNc of mice by 196.37 ± 17.39% (*p *<* *0.001), while the following exposure to MPTP+*miR-132-3p* antagomir repressed the level of *miR-132-3p* (*p* < 0.01, 125.59 ± 12.67% vs 179.34 ± 14.34%; Fig. 7*A*). Immunohistochemistry results displayed that there were enhanced expression of GLRX in the MPTP+*miR-132-3p* antagomir group (*p *<* *0.01, vs the MPTP+antagomir NC group, 87.25 ± 12.57% vs 57.16 ± 6.28%) and decreased level of GLRX in the MPTP group by 53.47 ± 6.39% (*p* < 0.001, vs the saline group; Fig. 7*B*). FISH and immunofluorescence were applied to detect the expressions of miR-132-3p and GLRX in microglial cells. The results showed that miR-132-3p expression of SNc of mice in MPTP group was increased by 2.16 ± 0.36-fold, while GLRX expression was suppressed by 0.46 ± 0.11-fold when compared with Saline group. Comparisons between MPTP+miR-132-3p antagomir group and MPTP+antagomir NC group demonstrated that miR-132-3p expression was suppressed (miR-132-3p: 1.58 ± 0.27-fold vs 2.34 ± 0.38-fold) and GLRX expression was elevated (GLRX: 0.84 ± 0.18-fold vs 0.53 ± 0.10-fold) in miR-132-3p antagomir group (*p *<* *0.01; Fig. 7*C*,*D*). Co-location analysis showed miR-132-3p expression in microglial cells accounted for 40% of miR-132-3p expression in tissues, while the expression of GLRX in microglial cells accounted for 55% of GLRX expression in tissues (Fig. 7*C*,*D*). Additionally, immunofluorescence of Iba1 exhibited that the microglial activation was increased in MPTP group (619.74 ± 88.90 vs 174.83 ± 23.03 cells/mm^2^, *p *<* *0.001) and decreased in MPTP+*miR-132-3p* antagomir group (417.36 ± 74.78 vs 595.72 ± 78.02 cells/mm^2^; Fig. 7*E*, *p* < 0.01). Analysis of qRT-PCR showed that MPTP treatment potentiated the mRNA levels of inflammatory cytokines TNF-α (1.68 ± 0.29-fold), IL-1β (1.79 ± 0.28-fold), IL-6 (1.84 ± 0.32-fold) in brain tissues of mice (*p *<* *0.01), whereas MPTP+*miR-132-3p* antagomir diminished the expressions of TNF-α (1.26 ± 0.21-fold vs 1.76 ± 0.30-fold), IL-1β (1.22 ± 0.19-fold vs 1.72 ± 0.31-fold), IL-6 (1.34 ± 0.23-fold vs 1.81 ± 0.28-fold; Fig. 7*F*, *p* < 0.05). Then, the assessment of the dopaminergic neuron loss by immunofluorescence of tyrosine hydroxylase illustrated that tyrosine hydroxylase-positive neurons in SNc of mice were significantly decreased in the MPTP group (*p *<* *0.001, 155.83 ± 25.97 vs 621.37 ± 91.97 cells/mm^2^) and markedly increased in the MPTP+*miR-132-3p* antagomir group (401.72 ± 58.83 vs 177.83 ± 32.00 cells/mm^2^; Fig. 7*G*, *p* < 0.01). The rotarod test and open field test were used to observe the motor ability of mice. In the rotarod test, mice in the MPTP group showed poor balance and coordination by suppressing the time to 57.38 ± 12.37%, while mice in the MPTP+*miR-132-3p* antagomir group exhibited better balance and coordination (84.72 ± 15.46 vs 52.43 ± 9.27%; Fig. 7*H*, *p* < 0.01). In the open field test, MPTP injection suppressed the spontaneous locomotor activities (whole area: 53.27 ± 8.91%, central area: 42.35 ± 6.28%), whereas MPTP+*miR-132-3p* antagomir increased the spontaneous locomotor activities (whole area: 82.47 ± 13.67% vs 48.37 ± 7.21%; central area: 78.61 ± 11.59% vs 46.52 ± 7.38%; Fig. 7*H*, *p* < 0.01). The above results indicated that MPTP injection may induce dopaminergic neurodegeneration and neuroinflammation of mice, while depletion of *miR-132-3p* may ameliorate the dopaminergic neuron degeneration and neuroinflammation of PD mouse.

## Discussion

Neuroinflammation is a characteristic of neurodegenerative diseases, including PD, in which microglia confer pathogenic and exacerbating effects (Lull and Block, 2010; Zhao et al., 2020). Furthermore, the notable hallmark of PD is the degeneration of dopaminergic neurons in the SNc (Mead et al., 2017). Herein, BV-2 cells and SH-SY5Y were used in current study to explore the effect of *miR-132-3p* on inflammation of microglial cells and neuronal injury. We have reported that *miR-132-3p* is abnormally upregulated, a change positively connected with the inflammatory response of microglial cells. We demonstrated that the activated microglial cells caused by *miR-132-3p* leads to increased cell apoptotic rate and diminished viability of neuroblastoma cells. However, *miR-132-3p* was also reported to alleviate neuron apoptosis and impairments of learning and memory abilities in Alzheimer’s disease (Qu et al., 2021). Alzheimer’s disease and PD, both belonging to neuro-degenerative diseases: the former is a neurodegenerative brain pathology formed because of piling up of amyloid proteins, development of plaques, and disappearance of neurons (Tufail et al., 2021), while the latter is caused by the loss of dopaminergic neurons in the substantia nigra (Xu et al., 2021). On parallel, the treatment strategies between our two literatures were also different. In our study, LPS induced BV-2 cells were used as inflammatory cell models and the supernatant of BV-2 cell was co-cultured with SH-SY5Y cells. As for the PD rat models, MPTP treatment was given to rats for consecutively 5 d. This discrepancy may be explained by the difference on the disease background and treatment strategy. Similar to our detection, previous studies identified that miR-132-3p was one of the upregulated cimiRNAs in patients with major depression disorder (van den Berg et al., 2020) and was found to be elevated in the serum of patients with mild cognitive impairment (Xie et al., 2015). In addition, we also revealed that GLRX suppresses activation of microglial cells and ameliorates neuronal injury caused by *miR-132-3p*. Finally, we found that MPTP-induced neuroinflammation and degeneration of dopaminergic neurons in PD mouse models are dramatically attenuated after *miR-132-3p* downregulation. Thus, our study not only uncovered novel roles for *miR-132-3p* and GLRX in the pathologic abnormalities related to PD but also identified their potential application in the treatment for PD.

Microglial cells are macrophages residing in the brain, which originate from early erythro-myeloid precursors in the embryonic yolk sac (Kanthasamy et al., 2019). Activated microglial cells at the inflammation site promote the release of inflammatory cytokines, thereby intensifying the inflammatory response through activation and recruitment of other cells to the brain lesion (Kim and Joh, 2006). Accumulating evidence proposed that in the process of PD, microglial cells are activated, and then trigger the secretion of a variety of proinflammatory factors, including IL-6, IL-1β, and TNF-α (Guan et al., 2017). In an attempt to elucidate the mechanism by which *miR-132-3p* accelerates the progression of PD, we first investigated whether *miR-132-3p* affects the activation of microglial cells. Initially, remarkable high expression pattern of *miR-132-3p* was noticed in midbrain tissues from patients with PD rather than tissues of healthy controls. To this end, LPS was applied to simulate the inflammatory response in BV-2 cells. Herein, results of gain-and loss-of-function experiments confirmed that *miR-132-3p* might likewise contribute to the activation of microglial cells. In our study, knock-down of miR-132-3p can suppress the release of inflammatory cytokines, including TNF-α, IL-1β, and IL-6, while overexpression of miR-132-3p can promote the inflammatory response in BV-2 cells, those results indicated that miR-132 as a driver of microglia proinflammatory responses. Of note, there has been relevant evidence supporting our findings that *miR-132* confers a pivotal role in intracerebral hemorrhage by regulating inflammation, which is evident from the activation state of microglial cells and the expression of proinflammatory cytokines (Zhang et al., 2017). Furthermore, by using CCK-8 assay and flow cytometry, we discovered that LPS can suppress survival rate of microglial cells and increase cell apoptosis, while further treatment by *miR-132-3p* knock-down partially reverse the LPS induced cell apoptosis and elevate cell survival rate to certain extent. Further measurement showed that activated microglial cells by *miR-132-3p* may lead to neuronal injury, as evidenced by reinforced cell apoptotic ability and reduced the proliferative ability of SH-SY5Y cells after SH-SY5Y cells were cultured with the conditioned medium of BV-2 cells which were transfected with *miR-132-3p* mimic, which highlighted the role of *miR-132-3p* in activation of microglial cells and neuronal injury. Interestingly, former work described that the dysregulation of *miR-132* leads to the occurrence and exacerbation of PD (Qian et al., 2017). The BACE1-AS/*miR-132-3p* axis is responsible for the berberine-mitigated neuronal injury in Alzheimer’s disease (Ge et al., 2020). Therefore, *miR-132-3p* may exert a negative effect on PD by inducing neuroinflammation and neuronal injury.

Subsequently, we are prompted to further look into the molecular actions of *miR-132-3p* in regulating PD by investigating the downstream target. Based on the comprehensive analysis from StarBase, dual-luciferase reporter assay and RIP assay, we identified GLRX as a direct target of *miR-132-3p*. GLRX is an indispensable thioltransferase whose main function is to remove protein glutathionylation (Burns et al., 2020). Herein, the lowly expressed GLRX was observed in the midbrain tissues of PD patients. Furthermore, analyses of qRT-PCR, Western blotting, ELISA, CCK-8 assay and flow cytometry elaborated that *miR-132-3p* interfered with microglial activation and neuronal injury by targeting GLRX. Our data are in agreement with the earlier findings showing that enhancement of GLRX activity in these brain cells would impede the progression of PD (Gorelenkova Miller and Mieyal, 2019). MPTP is a neurotoxin that results in a profound reduction of striatal dopamine levels and specific loss of dopaminergic neurons in animals (Lee et al., 2019). To further shed light into the relationship between *miR-132-3p*/GLRX and PD, we used a mouse model of PD stimulated by MPTP. Consistently, mice received MPTP injection had increased inflammatory cytokine release and decreased TH positive neurons, indicating the neuron loss in MPTP-treated mice. On parallel, elevated *miR-132-3p* expression and decreased expression of GLRX were also observed in mice in MPTP group, suggesting the possible implication of *miR-132-3p* in neuron loss of PD mouse. Here, we showed that *miR-132-3p* downregulation in PD mouse induces alterations in GLRX expression, and *miR-132-3p* was responsible for the inflammatory response of brain tissues of PD mouse models by modulating GLRX. Additionally, immunofluorescence of Iba1 on detection of microglial activation and immunofluorescence of tyrosine hydroxylase on assessment of dopaminergic neuron loss revealed that depletion of *miR-132-3p* may alleviate MPTP-induced dopaminergic neurodegeneration and neuroinflammation of PD mouse models. Simultaneously, these findings were further supported by the rotarod test and open field test.

In conclusion, our data suggest that the deficiency of *miR-132-3p* contributes to ameliorated PD. *MiR-132-3p* enhances the activation of microglia cells and promotes the release of inflammatory cytokines by targeting GLRX to exert toxic effect on neurons. These findings suggest that targeting neuroprotective pathways controlled by *miR-132-3p* may represent a potential therapeutic intervention strategy for PD therapy. Further work is required to ascertain whether the protection from PD observed here by silencing of the *miR-132-3p* is exerted by GLRX, inhibition of microglial activation and dopaminergic neuron loss or perhaps via modulation of other pathways.

## Synthesis

Reviewing Editor: Mikko Airavaara, Helsingin yliopisto Decisions are customarily a result of the Reviewing Editor and the peer reviewers coming together and discussing their recommendations until a consensus is reached. When revisions are invited, a fact-based synthesis statement explaining their decision and outlining what is needed to prepare a revision will be listed below. The following reviewer(s) agreed to reveal their identity: NONE.

The manuscript “MiR-132-3p promotes neuroinflammation and dopaminergic neurodegeneration by suppressing GLRX"is scientifically sound and well written and the reviewers have found it to very high quality. Authors noted that miR-132-3p promotes the activation of microglial cells and facilitates the release of inflammatory cytokines by targeting GLRX, which leads to neurodegeneration of dopamine neurons in the midbrain. On the other hand, depletion of miR-132-3p relieved the dopaminergic neuron degeneration and neuroinflammation in PD mouse model.The techniques and methodology used in the study are high standards, with all appropriate controls, stereological quantification etc. The data is well presented, with individual data points on graphs and corresponding western blot images. It’s also noteworthy that authors validated most of their results by at least 2 different methods (e.g. data from qPCR is validated through other measurements (western blot or ELISA), viability is measured by biochemical assay and flow cytometry). Additionally, authors elegantly validate their findings e.g. through GLRX transfection rescue experiments (FIG 6). Therefore the conclusions are well supported.

Although this study will progress the knowledge of the field, authors need to address following major concerns before its publication.

1. In line 338 authors write: RIP experiment was applied to verify the binding of miR-132-3p to GLRX mRNA. However, the RIP experiment, while very valuable, gives rather indirect support rather than hard evidence since both GLRX mRNA and miR-132-3p could be bound to different targets. Even after LPS treatment increase in Ago2 bound GLRX might not be related to an increase in miR-132-3p but changes in other miRs. RIP experiment after utilizing antago-miR/miR inhibitors or miR mimics would be more definitive. However, it will be enough if authors just clearly state that RIP data might be correlational.

2. What is miR inhibitor - it should be better explained.

3. Other studies show that miR-132-3p has antiapoptotic and protective action in neurodegenerative disorders [Qu et al 2021], how do authors respond to such strikingly different results?

See: Qu J, Xiong X, Hujie G, Ren J, Yan L, Ma L. MicroRNA-132-3p alleviates neuron apoptosis and impairments of learning and memory abilities in Alzheimer’s disease by downregulation of HNRNPU stabilized BACE1. Cell Cycle. 2021 Sep 29:1-12. doi: 10.1080/15384101.2021.1982507. Epub ahead of print. PMID: 34585626.

4. Why authors think GLRX as a potential downstream target of miR-132-3p? Authors need to give rationale for considering GLRX as a selective downstream target of miR-132-3p, if previously studied

5. In figure 7C&D, it would be interesting to know what proportion of miR-132-3p colocalizes with Iba1 and GLRX. This will give an idea of efficiency of miR-132-3p as a microglia activator.

6. From the data presented in Fig 5, miR-132-3p inhibitors increases the expression of GLRX; thus, conforming that at the baseline condition GLRX mRNA/protein expression is under negative regulation of miR-132-3p. However, authors need to discuss how miR-132-3p mimic reduces the GLRX levels.

7. From the previous literature, the etiology of PD is largely associated with neuronal cell death due to α-synuclein aggregation, mitochondrial dysfunction and noxious oxidant stress. However, it is not clear, how authors think, miR-132-3p could be triggered to mediate neuroinflammation related to the neuronal death. How authors controlled for the neurotoxin MPP+, a MPTP metabolite, associated effects? Please discuss these issues.

8. The expression pattern of Iba1 looks different in figure 7 C vs E. Please clarify the discrepancy.

9. Authors need to explain procedure for the collection of the midbrain tissue samples from the brains. How specifically midbrain tissue is isolated? In addition, the procedure for how tissue samples prepared for qRTPCR, Western blot, ELISA, etc. should be given.

10. Authors have injected five microliters of miR-132-3p antagomir suspension or antagomir NC suspension into the SNc in 5 min. In general, this is a large volume for the intracranial injection. Please confirm the volume injected.

11. How is relative time on the rod (%) and distance calculated for the rod test?

Minor text corrections:

12. In Line 34 authors mention among PD symptoms tremor - the PD patient show particularly resting tremor.

13. Also in same sentence (lines 33-36) the authors write “... postural instability difficulty with walking and gait” the wording and punctation seem to be not quite correct here and the authors should define some characteristics of Parkinsonian gait.

14. In line 50: the authors state that human geome encodes about 1500miRNA, this nuber might be slightly higher actually (∼1900 miRNA in miRbase); please refer to newest literature on the data.

15. Line 264: its better to use ’incubated’ instead of ’baked’. Give the % of xylene used.

16. Line 267, 286: Give the % of normal goat serum used.

17. Line 555: Please confirm if this statement is correct “MiR-132-3p enhances the activation of dopaminergic neurons and promotes the release of inflammatory cytokines by targeting GLRX to exert toxic effect on neurons.” Do you want to mean “MiR-132-3p enhances the activation of microglia...”?

18. Authors should clearly label the co-treatments groups in figure 7 for a better understanding of the reviewers.
